# Pulmonary Hypertension Complicating Fibrosing Mediastinitis

**DOI:** 10.1097/MD.0000000000001800

**Published:** 2015-11-06

**Authors:** Andrei Seferian, Alexandru Steriade, Xavier Jaïs, Olivier Planché, Laurent Savale, Florence Parent, David Amar, Roland Jovan, Elie Fadel, Olivier Sitbon, Gérald Simonneau, Marc Humbert, David Montani

**Affiliations:** From the University Paris-Sud, Faculté de Médecine (AS, AS, XJ, OP, LS, FP, DA, RJ, EF, OS, GS, MH, DM); AP-HP, Centre de Référence de l’Hypertension Pulmonaire Sévère, Département Hospitalo-Universitaire (DHU) Thorax Innovation (TORINO), Service de Pneumologie, Hôpital de Bicêtre, Le Kremlin Bicêtre (AS, AS, XJ, LS, FP, DA, RJ, OS, GS, MH, DM); UMR_S 999, University Paris–Sud; INSERM; Laboratoire d’Excellence (LabEx) en Recherche sur le Médicament et l’Innovation Thérapeutique (LERMIT), Centre Chirurgical Marie Lannelongue, Le Plessis Robinson (AS, AS, XJ, LS, FP, DA, RJ, EF, OS, GS, MH, DM); AP-HP, Service de Radiologie, Hôpital Bicêtre, Le Kremlin-Bicêtre (OP); and Centre Chirurgical Marie Lannelongue, Service de Chirurgie Thoracique, Le Plessis Robinson, France (EF).

## Abstract

Fibrosing mediastinitis is caused by a proliferation of fibrous tissue in the mediastinum with encasement of mediastinal viscera and compression of mediastinal bronchovascular structures. Pulmonary hypertension (PH) is a severe complication of fibrosing mediastinitis caused by extrinsic compression of the pulmonary arteries and/or veins.

We have conducted a retrospective observational study reviewing clinical, functional, hemodynamic, radiological characteristics, and outcome of 27 consecutive cases of PH associated with fibrosing mediastinitis diagnosed between 2003 and 2014 at the French Referral Centre for PH.

Fourteen men and 13 women with a median age of 60 years (range 18–84) had PH confirmed on right heart catheterization. The causes of fibrosing mediastinitis were sarcoidosis (n = 13), tuberculosis-infection confirmed or suspected (n = 9), mediastinal irradiation (n = 2), and idiopathic (n = 3). Sixteen patients (59%) were in NYHA functional class III and IV. Right heart catheterization confirmed moderate to severe PH with a median mean pulmonary artery pressure of 42 mm Hg (range 27–90) and a median cardiac index of 2.8 L/min/m^2^ (range 1.6–4.3). Precapillary PH was found in 22 patients, postcapillary PH in 2, and combined postcapillary and precapillary PH in 3. Severe extrinsic compression of pulmonary arteries (>60% reduction in diameter) was evidenced in 2, 8, and 12 patients at the main, lobar, or segmental levels, respectively. Fourteen patients had at least one severe pulmonary venous compression with associated pleural effusion in 6 of them. PAH therapy was initiated in 7 patients and corticosteroid therapy (0.5–1 mg/kg/day) was initiated in 3 patients with sarcoidosis, with 9 other being already on low-dose corticosteroids. At 1-year follow-up, 3 patients had died and among the 21 patients evaluated, 3 deteriorated, 14 were stable, and only 4 patients with sarcoidosis improved (4 receiving corticosteroids and 1 receiving corticosteroids and PAH therapy). Survival was 88%, 73%, and 56% at 1, 3, and 5 years, respectively.

We found no clear clinical improvement with the use of specific PAH therapy. Corticosteroid therapy may be associated with clinical improvement, in some patients with fibrosing mediastinitis due to sarcoidosis. Although never performed for this indication, lung transplantation may be proposed in eligible patients with severe PH and fibrosing mediastinitis.

## INTRODUCTION

Pulmonary hypertension (PH) is a progressive, severe hemodynamic disorder, which can be potentially fatal if left untreated. It is defined by an elevation of the pulmonary artery pressure above 25 mm Hg that leads to right ventricular failure. Assessment by right heart catheterization (RHC) is mandatory for diagnosis and multiple tests are used to classify PH in 1 of the 5 clinical groups.^1,2^ Group 1 PH corresponds to pulmonary arterial hypertension (PAH) which can be idiopathic, heritable, induced by drugs or toxins, and associated with connective tissue disease, HIV infection, congenital heart disease, or portal hypertension.^2^ A variety of hematologic, systemic, or metabolic disorders associated with PH by unclear and/or multifactorial mechanisms form group 5 of the classification. Pulmonary hypertension complicating fibrosing mediastinitis has been classified in group 5.^3^

Fibrosing mediastinitis is a rare, benign, but potentially lethal disorder caused by a proliferation of fibrous tissue in the mediastinum with encasement of the mediastinal viscera and extrinsic compression of mediastinal bronchovascular structures, which leads to a progressive, insidious disease with variable natural history.^4,5^ Fibrosing mediastinitis is usually associated with a history of granulomatous disease such as sarcoidosis, tuberculosis, or histoplasmosis.^6–8^ The most important series of cases describe American patients with fibrosing mediastinitis complicating histoplasmosis.^6,9,10^ Reports also point out to a possible association with other fungal infections including *aspergillosis, blastomycosis*, *mucormycosis, cryptococcosis,* or infection with *Wuchereria bancrofti*.^11–15^ Fibrosing mediastinitis has also been reported in the context of autoimmune disease (rheumatoid arthritis and systemic lupus erythematosus),^15^ Behçet disease,^14^ mediastinal radiation,^15,16^ and therapy with methysergide.^17^ Lastly, an idiopathic, nongranulomatous form has been described.^18,19^

Clinical presentation depends largely upon which structures of the mediastinum are affected, with cough, chest pain, and dyspnea being the main symptoms.^20^ Typical complications include superior vena cava syndrome, large airways compression which can lead to post obstructive pneumonia or atelectasis, bronchial erosion by calcific lymph nodes, oesophageal compression or pulmonary artery, and/or vein compression.^10,21,22^ Patients with compression of pulmonary vessels can have hemoptysis due to bronchial artery hypertrophy, which is frequently observed in fibrosing mediastinitis.^9,15^ Imaging studies and suggestive clinical context are usually sufficient to confirm the diagnosis. Diagnosis may be challenging in the context of PH because pulmonary arterial compression PH and right heart failure are considered to be an important cause of morbidity and mortality in fibrosing mediastinitis.^23,24^

In this study, we report the experience of the French Referral Centre for Pulmonary Hypertension (Université Paris Sud, Hôpital Bicêtre, Assistance Publique Hôpitaux de Paris, Le Kremlin Bicêtre, France) in the diagnosis, management and outcomes of consecutive patients with PH complicating fibrosing mediastinitis.

## METHODS

### Subjects

We have conducted a retrospective observational study reviewing all incident cases from the French PH Registry of confirmed precapillary PH among patients in whom the cause was fibrosing mediastinitis. This Registry was established in accordance with French bioethics laws (*Commission Nationale de l’Informatique et des Libertés*), and all patients gave their informed consent. Diagnosis of PH defined as mean pulmonary arterial pressure (mPAP) ≥ 25 mm Hg was confirmed in all patients by RHC.^1^ Routine evaluation at baseline included medical history, physical examination, echocardiography, contrast-enhanced high-resolution computed tomography (HRCT) of the chest, ventilation/perfusion (V/Q) lung scan, pulmonary angiography, abdominal ultrasound, autoimmunity screening, and HIV serology. For the present report, all data were reviewed and the diagnosis of PH complicating fibrosing mediastinitis was confirmed after a multidisciplinary meeting including senior pulmonologists (AS, XJ, LS, FP, OS, GS, MH, DM), radiologists (OP), and thoracic surgeons (EF). The cause of fibrosing mediastinitis was determined based on patient's medical history. Confirmed tuberculosis as the cause of fibrosing mediastinitis was defined as a definitive history of positive culture for *Mycobacterium tuberculosis* or history of treatment for tuberculosis. Tuberculosis was considered as suspected in patients with compatible clinical history of tuberculosis, originating from endemic countries and compatible parenchymal posttuberculosis sequellae on HRCT of the chest. A diagnosis of sarcoidosis required histological evidence of noncaseating granulomas in lymph nodes or bronchial biopsies.

### Hemodynamic Assessment

Right atrial pressure (RAP), systolic, diastolic and mean PAP, pulmonary artery wedge pressure (PAwP), and mixed venous saturation (SvO_2_) were measured during RHC. Cardiac output (CO) was measured by the standard thermodilution technique. The cardiac index was calculated as the CO/body surface area, expressed in L/min/m^2^. Pulmonary vascular resistance (PVR) was calculated as (mPAP-PAwP)/CO, expressed in Wood units (WU). Acute vasodilator testing was performed during RHC with inhaled nitric oxide, as previously described.^25,26^ Precapillary PH was retained if PAwP was ≤ 15 mm Hg, and postcapillary PH was retained if PAwP was > 15 mm Hg. In the presence of elevated PAwP, a gradient between diastolic PAP and the PAwP ≥ 7 mm Hg was used to establish a diagnosis of combined postcapillary PH with a precapillary component.^3,27^

### Clinical and Functional Assessment

Dyspnea was assessed at PH diagnosis by New York Heart Association (NYHA) functional class. A nonencouraged 6-min walk test according to recommendations was performed.^28,29^ Arterial blood gases and pulmonary function tests were recorded, including partial pressure of arterial oxygen (PaO_2_) and partial pressure of arterial carbon dioxide (PaCO_2_), the forced expiratory volume in 1 second (FEV1), the forced vital capacity (FVC), the total lung capacity (TLC), and diffusing capacity for carbon monoxide (DLCO). An obstructive and restrictive pattern was defined as a FEV1/FVC < 0.7 and TLC < 80% predicted respectively, whereas a mixed pattern was a combination of the both.

### Radiologic Findings

All HRCT of the chest, V/Q lung scans and pulmonary angiography, were reviewed by experienced PH specialists and radiologists (AS, OP, DM). Arterial, venous, or bronchial compressions were evaluated by measuring the reduction in diameter of their lumen in the axial plane. For bronchus and pulmonary arteries, compressions were analyzed at the main pulmonary, lobar, and segmental levels. For pulmonary veins, compressions were evaluated at the level of the main superior and inferior left and right pulmonary veins. The threshold of a reduction of 60% in diameter of pulmonary arteries and bronchi with respect to upstream or downstream diameter was chosen to define severe stenoses. When a lobar artery (or bronchus) appeared severely stenosed over all its length, the downstream diameter used to confirm this severe stenosis was estimated from the distal normal diameter of its segmental branches. If no distal normal diameter could be found because all these branches were also severely affected over all their length or occluded, the lobar artery or bronchus was considered severely stenosed. For pulmonary veins, severe stenoses were defined as a reduction in diameter of >60% when compared to the normal diameter of the vein upstream or downstream of the stenosis. When no normal diameter could be found, stenoses were estimated as an average of the stenoses of its immediate tributary veins. Other parameters on HRCT were evaluated including the lung parenchyma, the presence of pleural or pericardial effusion, the oesophagus, the superior vena cava, and the retroperitoneal space.

### Statistical Analysis

All results are presented as median (range). Information on survival was available for all patients on December 31st 2014. Survival analysis was performed by using the Kaplan–Meier survival curve by using the GraphPad Prism 6 software (La Jolla, CA).

## RESULTS

### Subjects

Between 2003 and 2014, 31 patients with fibrosing mediastinitis and possible PH were evaluated at the French PH Referral Central. Of note, 4 patients (1 silicosis and 3 tuberculosis) had no PH, but mild elevation in mPAP ranging between 20 and 24 mm Hg and therefore were not included in our analysis. Thus, PH was confirmed by RHC in 27 patients. Anthropometric data, clinical, functional, and hemodynamic parameters of patients with PH complicating fibrosing mediastinitis are summarized in Tables 1 to 3. There were a total of 14 men and 13 women with a median of age of 60 years (range 18–84). Fifteen patients were Caucasians and 12 were Africans. The mean delay between the onset of symptoms and PH diagnosis was almost 3 years. Thirteen patients developed fibrosing mediastinitis in the context of sarcoidosis, 7 of them having stage 4 disease. In 9 patients, history of tuberculosis was suspected (n = 6) or confirmed (n = 3). Irradiation of the mediastinum was incriminated in 2 patients. One patient received radiotherapy of the mediastinum 30 years before for Hodgkin lymphoma, whereas the second one was irradiated for a locally advanced breast carcinoma 15 years prior to PH diagnosis. For the remaining 3 patients, no cause of fibrosing mediastinitis was found and fibrosing mediastinitis was considered idiopathic.

**TABLE 1:**
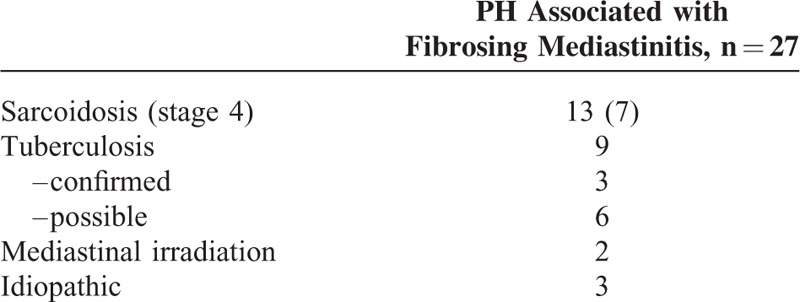
Etiologies of Fibrosing Mediastinitis

**TABLE 2:**
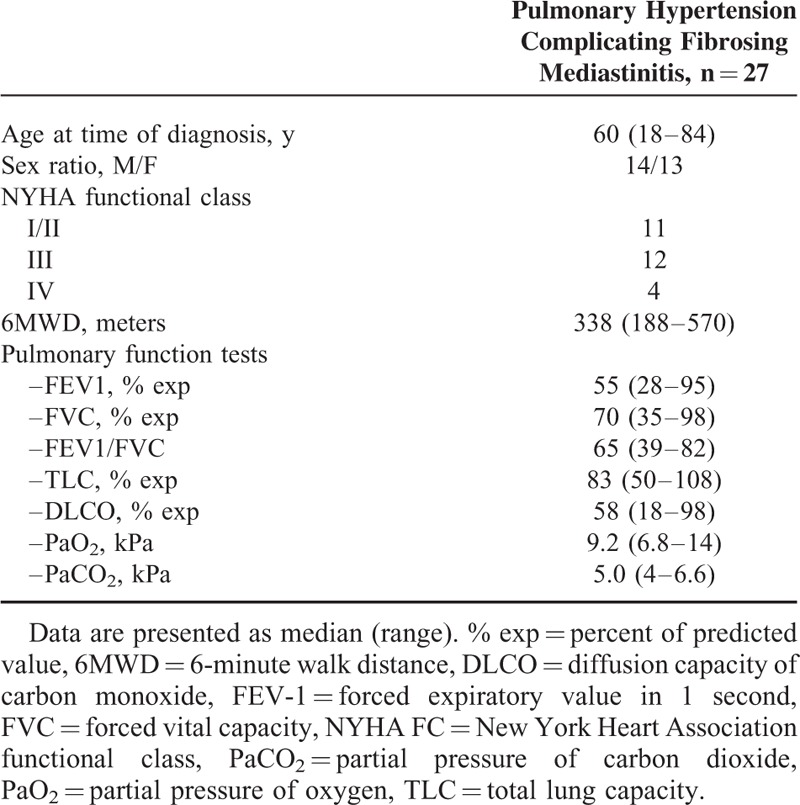
Characteristics of Patients With Pulmonary Hypertension Complicating Fibrosing Mediastinitis

**TABLE 3:**
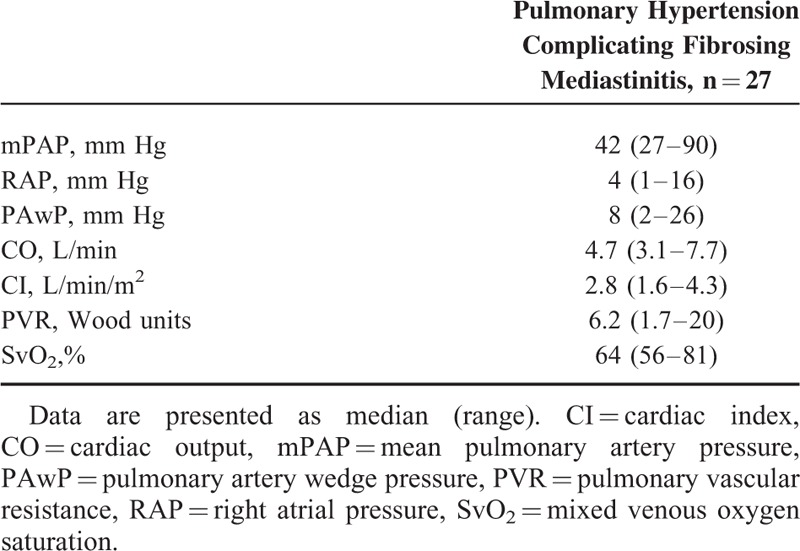
Hemodynamics of Patients with Pulmonary Hypertension Complicating Fibrosing Mediastinitis

### Clinical and Functional Characteristics

Sixteen patients (59%) had severe exercise limitation and were in NYHA functional classes III and IV. The median 6-min walking distance (6MWD) was 338 m (range 188–577). No patient had clinical superior vena cava syndrome and 6 patients had history of mild hemoptysis. Pulmonary function tests confirmed an obstructive pattern in 15 patients, a restrictive pattern in 3 patients, a mixed pattern in 3 patients, and only 6 patients had normal lung volumes and flows. The median TLC was 83% of theoretical values (range 50–108), the median FEV1/FVC was 65% (range 39–82), the median FVC was 70 (range 35–98), whereas the median FEV1 was 55% (range 28–95). Diffusion capacity for carbon monoxide was reduced at 58% of theoretical values (range 18–98). Arterial blood gases abnormalities were common on room air (median PaO_2_ of 9.2 kPa [range 6.8–14] and median PaCO_2_ of 5 kPa [range 4–6.6]).

### Hemodynamics

Right heart catheterization confirmed moderate to severe PH with a median mPAP of 42 mm Hg (range 27–90), a median cardiac index of 2.8 L/min/m^2^ (range 1.6–4.3), and a median RAP of 4 mm Hg (min-max 1–19). A total of 22 patients had precapillary PH, 2 had postcapillary PH, whereas 3 had combined postcapillary PH with a precapillary component based on an elevated gradient between diastolic PAP and PAwP. Median PVR was 6.2 WU (range 1.7–20 WU).

### Radiologic Findings

**FIGURE 1:**
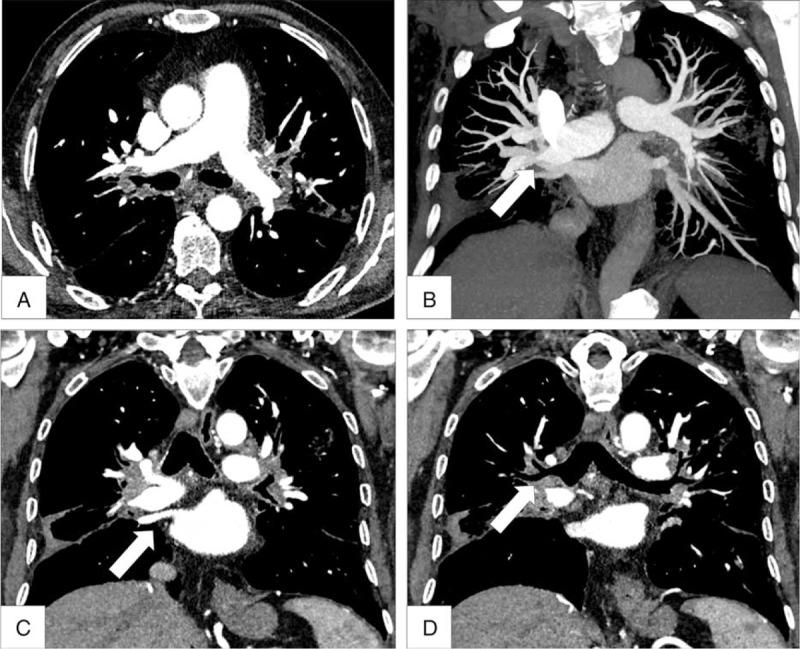
Contrast-enhanced high-resolution computed tomography of the chest of a patient with pulmonary hypertension complicating fibrosing mediastinitis. Panels A and B: Cross-sectional and coronal views showing extrinsic bilateral main pulmonary arteries compression by fibrosing mediastinitis, Panel C: Coronal view showing extrinsic venous compression, Panel D: Coronal view showing bilateral bronchial compression.

Involvement of the pulmonary arteries, pulmonary veins, or bronchi was assessed on HRCT of the chest in all patients. Radiological findings of patients with PH complicating fibrosing mediastinitis are presented in Table 4. Figure 1 illustrates a typical case with pulmonary arterial, bronchial, and pulmonary venous compression. Calcified lymph nodes of >1 cm were found in 15 of the 27 patients (55%). At the pulmonary arterial level, fibrosing mediastinitis severely compressed (>60% of the arterial lumen) main pulmonary, lobar, and segmental arteries in 2, 8, and 12 patients, respectively. Compression of at least 1 pulmonary vein (>60% of the venous lumen) was found in 14 of the 27 patients. Severe bronchial compression (>60% of the bronchial lumen) was found at the lobar and segmental levels in 8 and 12 patients, respectively. Atelectasis was present in 10 patients (37%), all of them having bronchial compression at the lobar and/or segmental levels. Pulmonary parenchymal abnormalities including ground glass opacities, septal lines, peribronchovascular thickening, and micronodules were observed in 18 (66%), 6 (22%), 11 (40%), and 5 (19%) patients, respectively. Fourteen patients had associated pulmonary fibrosis and 11 patients (40%) had severe pulmonary fibrosis (2 patients with previous history of radiotherapy, 7 with stage 4 sarcoidosis, and 2 with a history of tuberculosis). High-resolution computer tomography of the chest showed pleural effusion in 5 patients (19%), 3 of them having a severe venous compression in at least 1 pulmonary vein. Pericardial effusion was found in 4 patients (15%), 3 with sarcoidosis, and 1 with suspected history of tuberculosis. Retroperitoneal fibrosis was not observed in any of the 27 patients. A compression of the superior vena cava defined by a reduction of at least 60% of the superior vena cava lumen was found in 2 patients. Hypertrophic systemic bronchial arteries were identified in 6 of the 27 patients.

**TABLE 4:**
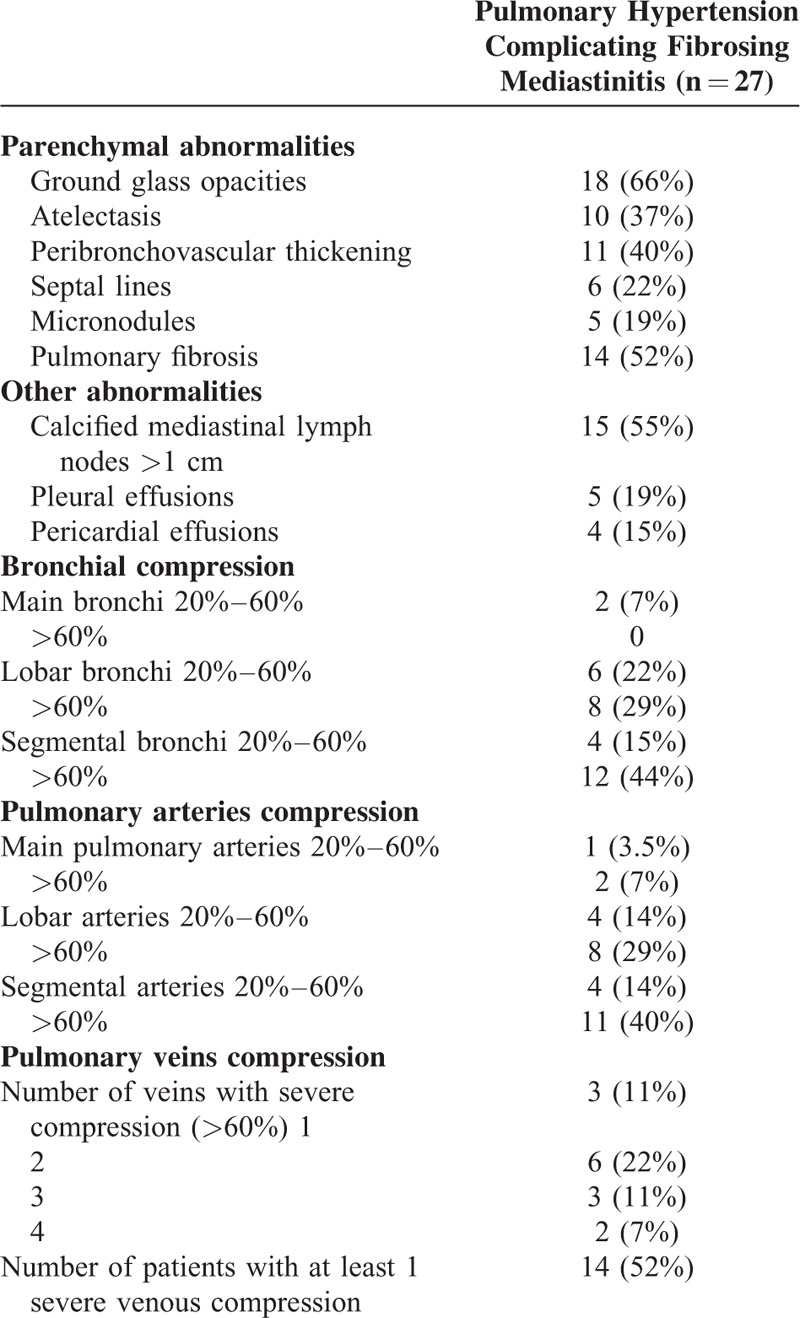
Radiological Findings on High-Resolution Computed Tomography in Patients with Pulmonary Hypertension Complicating Fibrosing Mediastinitis

V/Q lung scans were performed in 22 of the 27 patients, showing ventilation defects in 14 (63%), and segmental perfusion defects in 21 (95%). Fifteen patients (68%) had unmatched perfusion defects. Six patients underwent pulmonary angiography to exclude chronic thromboembolic pulmonary hypertension (CTEPH) and confirm extrinsic compression or encasement of pulmonary arteries.

### Initial Management

After diagnosis of PH complicating fibrosing mediastinitis, off-label PAH therapy was initiated in 6 patients with precapillary PH (4 with sarcoidosis, 1 with suspected tuberculosis, and 1 considered as idiopathic). There were no predefined criteria for the use of PAH therapies, as each case was evaluated by experts at the time of PH diagnosis and therapy was consensually initiated on a case per case basis. Treated patients received oral therapy with either endothelin receptor antagonists (ERA), phosphodiesterase 5 inhibitors (PDE5-i) or both. Corticosteroids were started at the dose of 0.5 to 1 mg/kg in 3 patients with fibrosing mediastinitis secondary to sarcoidosis. Nine other patients with fibrosing mediastinitis and sarcoidosis were already on corticosteroids at a mean dose of 20 mg/day (range 5–30 mg/d) at time of PH diagnosis. Concomitant use of PAH therapies and corticosteroids was recorded in 4 patients all of them with sarcoidosis. Anticoagulants were prescribed to 18 (67%) patients in order to reduce the risk of thrombosis, and 14 (52%) patients received oral diuretics to prevent fluid retention.

### Follow-up and Survival

**FIGURE 2:**
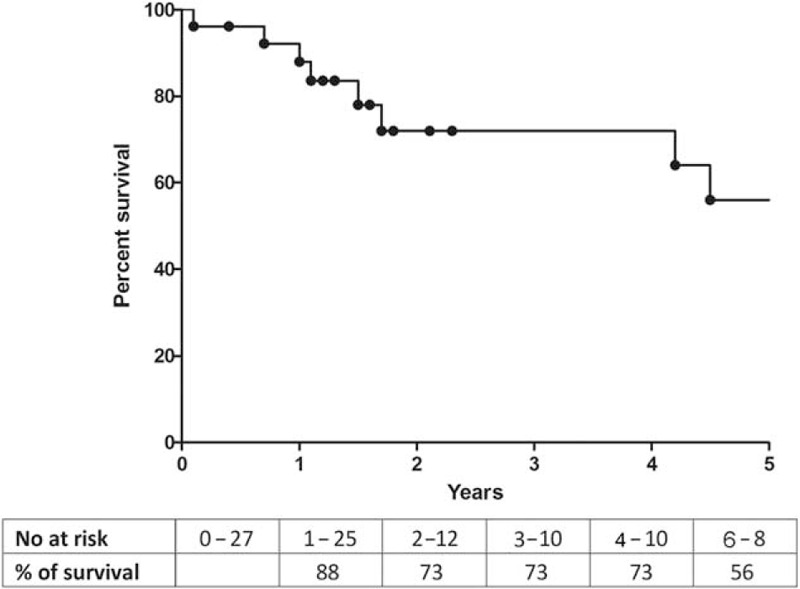
Overall 5-year survival of 27 patients with pulmonary hypertension complicating fibrosing mediastinitis.

**FIGURE 3:**
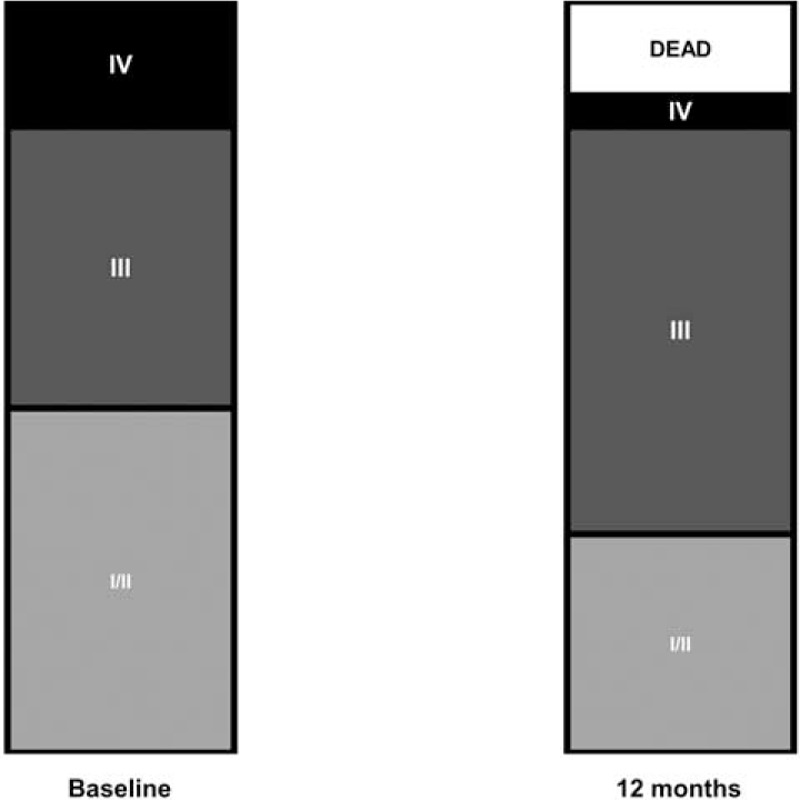
Comparison of New York Heart Association (NYHA) functional class at baseline and at 12 months (n = 25).

**FIGURE 4:**
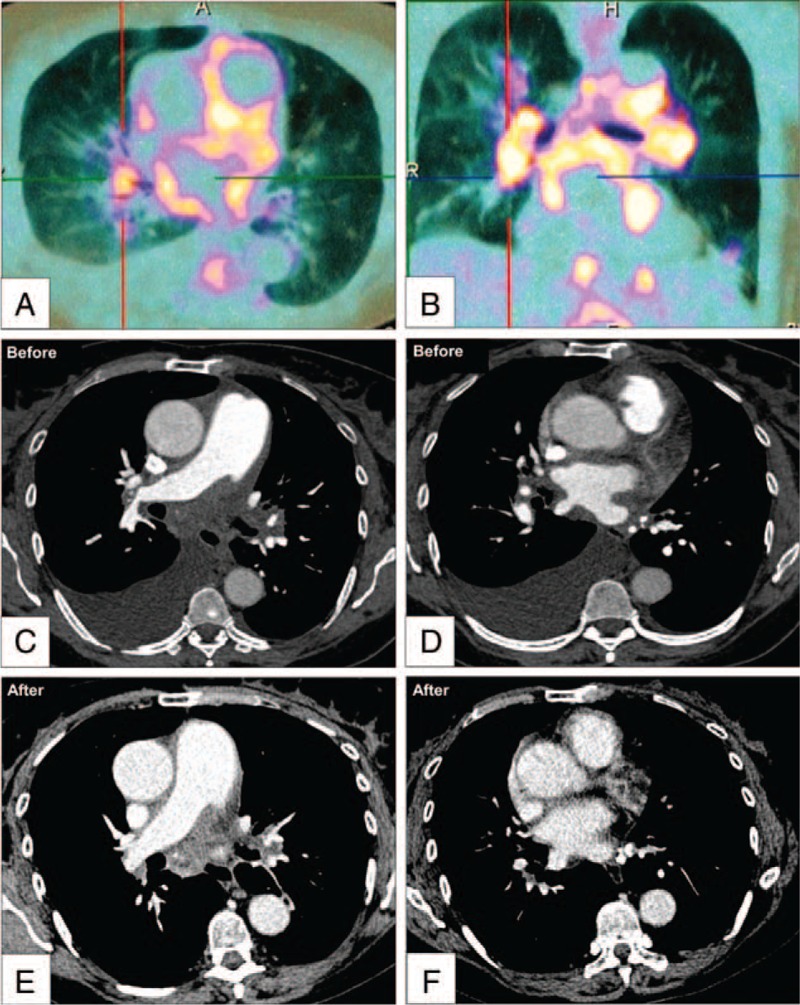
Panels A and B: cross-sectional and coronal views showing increased F-18 fluorodeoxyglucose uptake of the mediastinum in PET-scanning; Panels C and E: cross-sectional views showing extensive fibrosing mediastinitis with severe compression of the right pulmonary artery and veins and unilateral pleural effusion before corticosteroid therapy; Panels D and F: cross-sectional views showing a reduction in vascular and bronchial compression with the disappearance of the pleural effusion after corticosteroid treatment.

Median follow-up was 19 months (range 1–235). The survival analysis for all the patients is presented in Figure 2 showing 88%, 73%, and 56% survival rates at 1, 3, and 5 years respectively. A total of 8 patients died on follow-up, 5 with sarcoidosis, 1 with confirmed tuberculosis, and 2 with history of radiotherapy. Six patients died due to terminal right heart failure, whereas the rest of them due to acute respiratory failure complicating chronic respiratory failure. As not all the patients underwent hemodynamic assessment on follow-up, we used NYHA functional class as the main criteria to evaluate evolution of our patients. At 1 year of follow-up, 3 patients were dead and 21 patients were evaluated, showing clinical improvement in 4 patients, stability in 14 patients, and deterioration in 3 (Fig. 3). All the 4 patients with improved NYHA functional class had precapillary PH and sarcoidosis (2 with stage 4 disease and 2 with mild interstitial lung disease), all of them being on corticosteroids (3 on stable low dose and 1 initiated at 1 mg/kg/day 1 year before) and 1 also on PAH therapy. Right heart catheterization was performed in 3 of them confirming hemodynamic improvements with a reduction in PVR of 10%, 11%, and 75%, respectively. At time of diagnosis 1 of these patients with sarcoidosis underwent whole body positron emission tomography, which showed extensive metabolism of the mediastinum. This patient improved dramatically with corticosteroid therapy at a dose of 1 mg/kg (case presented in Fig. 4).

On long-term follow-up, none of the other patients improved NYHA functional class and 5 other patients died, whereas a total of 7 patients deteriorated at their last visit after more than 1 year.

## DISCUSSION

Fibrosing mediastinitis is an uncommon disorder characterized by inflammation and progressive fibrosis within the mediastinum.^9,30^ Pathophysiological mechanisms leading to fibrosing mediastinitis are largely unknown. The fibrotic process is thought to be a sequel of active granulomatous diseases, with immunogenic material causing an intense fibrotic reaction.^31^ The largest series of fibrosing mediastinitis were reported in the context of histoplasmosis.^6,9,10^ In this particular context, it has been demonstrated that tissues of fibrosing mediastinitis were characterized by an accumulation of CD20-positive B lymphocytes.^9^ An association between fibrosing mediastinitis and MHC class I antigen presentation molecules HLA-A2 has been reported, which supports the involvement of host-specific immune factors.^32^ The end of these processes is the development of nonspecific respiratory or extra-respiratory symptoms due to compression of the mediastinal bronchial and/or vascular structures.^9,12^

PH provoked by the extrinsic compression of the pulmonary arteries is considered as a rare but severe complication of fibrosing mediastinitis.^24^ To our knowledge we report in the present study the largest series of patients with fibrosing mediastinitis complicated by PH, all confirmed by RHC. Unlike previous American series, none of our patients had history of *Histoplasma capsulatum* infection.^6,9^ By contrast, sarcoidosis and tuberculosis are relatively common causes of thoracic granulomatous disease in Africans and Europeans and were the leading causes of fibrosing mediastinitis in our series,^33^ confirming previous findings by Mole et al.^15^ Thoracic radiotherapy was found in a couple of cases, whereas only 3 patients had no obvious etiologies and were considered to have idiopathic fibrosing mediastinitis. The age of diagnosis of PH complicating fibrosing mediastinitis was 60 years in our cohort, older than the previously reported age in the largest American posthistoplasmosis cohort (median of 42 years).^9,24,34,35^ This difference may be explained at least in part by the differences in the causes of fibrosing mediastinitis.

**FIGURE 5:**
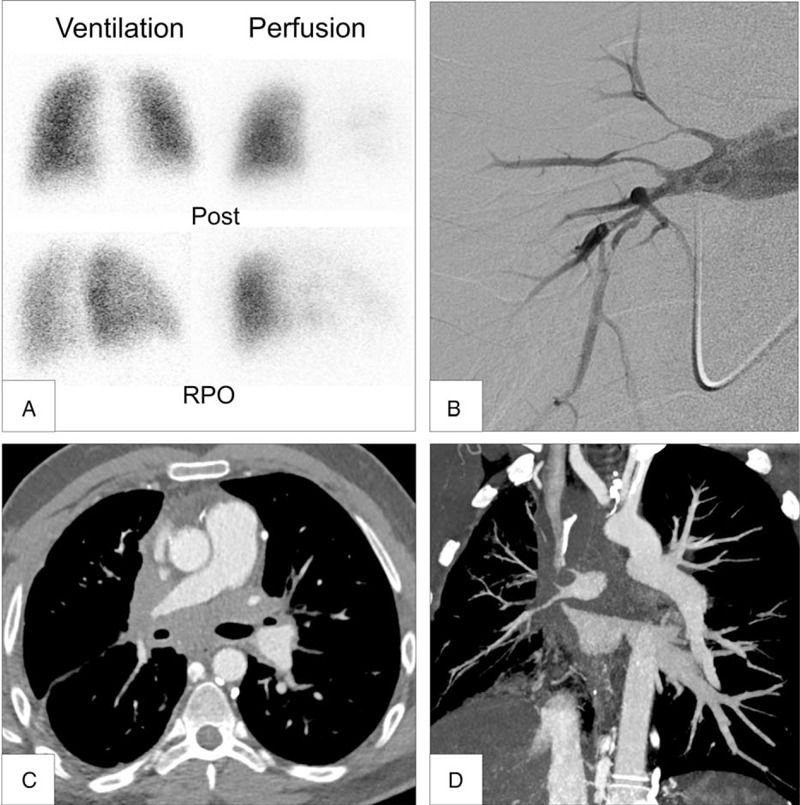
V/Q lung scan, contrast-enhanced high-resolution computed tomography and pulmonary angiography in a patient with pulmonary hypertension complicating fibrosing mediastinitis masquerading as chronic thromboembolic pulmonary hypertension. Panel A: V/Q lung scan showing multiple segmental perfusion defects in contrast with normal ventilation. Panels B: selective right pulmonary angiogram showing filiform right pulmonary arteries. Panels C and D: cross-sectional and coronal views of contrast-enhanced high-resolution computed tomography showing extrinsic pulmonary arterial compression by fibrosing mediastinitis. Post = posterior view on V/Q lung scan; RPO = right posterior oblic view on V/Q lung scan.

The majority of our patients had severe clinical and functional impairment with 59% of patients in NYHA functional class III or IV at PH diagnosis. The main reported symptom was exertional dyspnea and all patients were addressed to our PH clinic for the investigation of an increased estimated systolic pulmonary arterial pressure on echocardiography. For the vast majority of patients, fibrosing mediastinitis was not suggested at time of referral and the main suspected diagnosis was CTEPH because of mismatched segmental perfusion defects on V/Q lung scan. Diagnosis of fibrosing mediastinitis was based on HRCT of the chest and pulmonary angiography for the most difficult cases after adjudication by a panel of PH experts including pulmonologists, radiologists, and thoracic surgeons. Figure 5 shows a typical case of PH complicating fibrosing mediastinitis that could masquerade as CTEPH.

Right heart catheterization showed great disparities, some patients having mild PH and others severe hemodynamic impairment. Most of the patients (22/27) had pure precapillary PH but postcapillary PH was present in 5 patients (isolated in 2 and associated to precapillary PH in 3 patients). Severe pulmonary venous compression probably explained this postcapillary component in these 5 patients without comorbid cardiac disease. Pulmonary fibrosis was found in 14 of the 27 patients, 11 of them having a severe form. Most of these patients had either sarcoidosis with an important interstitial pulmonary disease or extensive sequelae of tuberculosis. Thus, we could not exclude that part of the increased PVR may be secondary to parenchymal involvement. It is worth mentioning that 3 of the 4 patients with pericardial effusions died on follow-up, which underlines that this specific PH severity factor may also play a prognostic role in some Group 5 PH patients. Pleural effusions were found in patients with at least 2 pulmonary veins severely occluded. Unlike previously reported symptoms in fibrosing mediastinitis associated with histoplasmosis, only 2 of our patients had radiological compression compatible with a superior vena cava syndrome but without any clinical manifestation. This difference in clinical presentation may be explained by the preferential localization of fibrosing mediastinitis, predominating in superior mediastinum for histoplasmosis and in medium mediastinum for patients who developed pulmonary vascular compression.

As the patients were evaluated over a large period of time (from 2003 to 2014) our initial management of PH and fibrosing mediastinitis was influenced by the available tools and our growing expertise. As part of the initial management, corticosteroids were newly introduced in only 3 patients, whereas 9 other patients were already on corticosteroids. They were used in patients with fibrosing mediastinitis associated with pulmonary sarcoidosis. Positron-emission tomography (PET-scanning) was performed at time of diagnosis in 3 patients with sarcoidosis showing activity of the disease and incited us to start corticosteroids in these patients. The initial result of the PET scanning was not a good predictor for response to corticosteroids as the clinical condition only improved in 1 of these patients. Such a resistance to corticosteroid therapy was also described by Peikert et al, in their cohort of fibrosing mediastinitis due to histoplasmosis ^9^ and may be explained by the advanced fibrotic lesions in which inflammation may have a lesser role. Taking into account these facts, the place of corticosteroid therapy in the management of patients with PH complicating fibrosing mediastinitis is still unclear and further studies are needed to better define their indications.

Off-label use of PAH therapy was based on hemodynamic severity. One treated patient died on follow-up, whereas the rest of them remained either stable or improved their NYHA functional class. However, we cannot issue formal recommendations on PAH drug use taking into account the heterogeneity of pulmonary arterial, venous, or lung parenchymal involvement. Use of PAH therapy must be carefully evaluated in these patients because these drugs may induce pulmonary edema in the case of pulmonary venous involvement or deteriorate arterial oxygen saturation in the case of severe parenchymal involvement. As the main mechanism of PH in these patients was compression of main pulmonary arteries, the rationale for using PAH therapies is weak in PH complicating mediastinal fibrosing.

Due to the extrinsic compression on pulmonary arteries and veins at different levels and the abnormalities of blood flow, one can suggest that there is an increased risk of thrombosis in these patients, which may incite the prescription of anticoagulants. However, hypertrophy of the bronchial arteries was found in 6 patients, 3 of them having history of mild hemoptysis. Therefore the benefit/risk ratio must be taken into account before initiating anticoagulation.

In the literature, there are few reports of using nonsurgical or surgical procedures to resolve superior vena cava, bronchial and pulmonary arterial or venous compressions.^9,24,34,35^ Peikert et al reported that balloon pulmonary artery angioplasty/stent, balloon pulmonary vein angioplasty, or bypass surgery for the superior vena cava syndrome were sometimes feasible in fibrosing mediastinitis.^9^ However, hemodynamic effects and long-term efficacy of these procedures are currently unknown. Up-to-date in literature there are no reports on lung transplantation in patients with fibrosing mediastinitis. In theory such an attitude may be indicated in the most severe forms, but the surgical procedure can be very complicated due to mediastinal adherences. It is important to mention that 1 of the patients with fibrosing mediastinitis associated with tuberculosis had a clinical superior vena cava syndrome that required a surgical by-pass between the brachiocephalic vein and the right atrium. Four years later, the patient started complaining of exertional dyspnea with a severe proximal bilateral pulmonary artery stenosis. A RHC was performed and found no PH, but a mild elevation of the mPAP of 24 mm Hg. Despite the absence of PH, but taking into account the clinical and radiological aspect we decided to perform 2 additional by-passes between the main pulmonary artery and the distal part of the right and left pulmonary arteries. The patient is still alive 13 years after the surgery and is asymptomatic with a normal echocardiography advocating maybe for a preventive surgery before the development of PH.

Rituximab has been proposed in 3 patients with fibrosing mediastinitis associated with histoplasmosis.^9,36^ By targeting surface antigen CD20, rituximab may eliminate B lymphocytes which infiltrate sheets or follicular structures associated with areas of fibrosis.^9,36^ It helped reduce lesion size and metabolic activity in the mediastinum in all 3 patients.^36^ However, further studies are needed to evaluate the potential impact of this therapy in these patients.

When compared with the only analysis performed up to date by Peikert et al, there is a clear difference in survival in our cohort.^9^ Having PH complicating fibrosing mediastinitis is associated with a poor prognosis (70% of our patients were alive 3 years after PH diagnosis as compared to >95% in the American cohort of histoplasmosis without PH). This difference could be explained by a more severe hemodynamic compromise at diagnosis unresponsive to medical therapy and also by the frequent severe parenchymal involvement observed in our patients with a history of sarcoidosis or tuberculosis.

Our study has several limitations. First, it is limited to the experience of a single national referral centre over a long period of time. Gradually, novel specific oral PAH therapies have become available and their use in clinical practice evolved in parallel. Another limitation is that the diagnosis of fibrosing mediastinitis was not confirmed histologically because mediastinoscopy or endoscopic bronchial ultrasound fiberoptic bronchoscopy with biopsies were considered as high-risk procedures in most PH patients. In almost all cases, use of corticosteroids was not incited by positive PET-scanning, as the availability of this investigation has also evolved recently. Last, some patients had no hemodynamic assessment on follow-up, and we cannot therefore discuss the progression of PH.

In conclusion, our series indicates that PH complicating fibrosing mediastinitis is a severe and complex condition with an important clinical and survival impact. Correct diagnosis of this association might be challenging but HRCT of the chest is helpful to characterize pulmonary arterial, venous, and bronchial compression and rule out CTEPH. The causes of fibrosing mediastinitis in France are different from the American cohorts, as sarcoidosis, tuberculosis, and mediastinal irradiation represent the vast majority of recognized cases. There was no clear clinical improvement with the use of PAH therapy and corticosteroids may be rarely associated with clinical improvement, in particular in active sarcoidosis. Further studies are needed to evaluate the impact of innovative therapies targeting the fibrotic process of the fibrosing mediastinitis and pulmonary angioplasty to tackle the consequences of arterial stenosis. Last, lung transplantation may be proposed in eligible patients with severe PH and fibrosing mediastinitis.
