# Pulmonary Hypertension Complicating Fibrosing Mediastinitis: Erratum

**DOI:** 10.1097/01.md.0000479539.61864.cd

**Published:** 2016-01-08

In the article “Pulmonary Hypertension Complicating Fibrosing Mediastinitis”,^1^ which appeared in Volume 94, Issue 44 of *Medicine*, Table 4 and Figure 2 were presented incorrectly. The article has since been corrected online.
